# Supplementary material for: Baculoviruses remodel the cytoskeleton of insect hemocytes to breach the host basal lamina
Source: Commun Biol. 2025 Feb 26;8:268. doi: 10.1038/s42003-025-07579-x (PMC11865517; doi:10.1038/s42003-025-07579-x)
Supplement: Supplementary file 2 — Supplementary Information [file 42003_2025_7579_MOESM2_ESM.pdf]

Supplementary Information of

Baculoviruses remodel the cytoskeleton of insect hemocytes to breach the host basal lamina

Authors: Ryuhei Kokusho*, Susumu Katsuma*

Affiliations:

Department of Agricultural and Environmental Biology, Graduate School of Agricultural and Life Sciences, the University of Tokyo; Tokyo, 113-8657, Japan.

*Corresponding authors. Email: rkokusho@g.ecc.u-tokyo.ac.jp (R.K.); skatsuma@g.ecc.u-tokyo.ac.jp (S.K.)

Supplementary Methods:

Western blotting

BmN cells were mock-infected or infected with WT, ARIF-GFP, or its partial deletion variants at a multiplicity of infection (MOI) of 5. They were collected at the indicated time points to detect the GFP-fused ARIF-1 protein. For time-course samples, the cells were homogenized in SDS-free RIPA buffer (150 mM NaCl, 50 mM Tris-HCl pH 8.0, 1% NP-40, 0.5 % sodium deoxycholate, cOmplete Mini Protease Inhibitor Cocktail (Roche)) and 2 × SDS sample buffer (4% SDS, 20% glycerol, 100 mM Tris-HCl pH 6.8, 0.05% BPB, 12% 2-mercaptoethanol) (1:1). For subcellular fractionation samples, the cells were fractionated using Subcellular Protein Fractionation Kit for Cultured Cells (Thermo Fisher Scientific) according to the manufacturer's protocol. Following an overnight incubation at room temperature, the proteins were separated on 10% SDS gels in the running buffer (25 mM Tris, 192 mM Glycine, 0.1% SDS) and transferred to the PVDF membrane as previously reported[1]. After blocking with 4% Block Ace (DS Pharma Biomedical, Japan), the membrane was incubated with the primary antibody (rabbit polyclonal anti-GFP antibody, 1:2,000; MBL, Japan; #598) in a combination of TBS-T buffer (20 mM Tris pH 8.0, 500 mM NaCl, 0.05% Tween 20) and 4% Block Ace (11:1). After incubation with the primary antibody overnight at 4°C, the membrane was washed three times with TBS-T and then incubated with the secondary antibody (HRP-conjugated goat anti-rabbit IgG, 1:5,000; Invitrogen; #656120) in TBS-T and Block Ace at room temperature for 1 h, followed by three TBS-T washes. Immobilon Western Chemiluminescent HRP Substrate (Millipore), the CCD imager LAS-1000 (Fujifilm, Japan), and Image Reader LAS-1000 Lite V1.31 software (Fujifilm, Japan) were used to detect chemical luminescence from antibody-stained proteins.

Immunostaining of virus-infected cultured cells

BmN cells were cultured on sterilized cover glasses on 6-well plates for immunostaining and infected with recombinant BmNPVs at an MOI of 5. The cells were fixed in 3.7% paraformaldehyde for 10 min, washed three times in 0.1% BSA/PBS, permeabilized in 0.5% Triton X-100 in 0.1% BSA/PBS for 10 min, washed three times in 0.1% BSA/PBS, and incubated overnight at 4°C with the primary antibody (rabbit polyclonal anti-GFP antibody, 1:400; MBL, Japan, #598). The cells were washed three times in 0.1% BSA/PBS and then incubated for 1 h with the secondary antibody (Alexa Fluor 488-conjugated goat anti-rabbit antibody, 1:400; Invitrogen, #11070). After three washes in 0.1% BSA/PBS, the cells were stained with -Cellstain- DAPI solution (1:500; DOJINDO,

Japan) and Rhodamine phalloidin (1:20; Invitrogen) in 0.1% BSA/PBS for 30 min. Subsequently, they were washed three times in 0.1% BSA/PBS and then sealed in 10 µL of ProLong Gold Antifade Reagent (Invitrogen). The samples were then examined using a confocal scanning laser microscope (Nikon C1Si) with the same settings as the observation of immunostained tissues. Unless otherwise specified, all the incubations were carried out at room temperature.

Survival curves of BmNPV-infected larvae

Newly-molted fourth-instar larvae were infected with BmNPVs (1×10^5 PFU/larva), observed every 6 h, and the death was counted. Survival curves were drawn using the Prism 8 software (GraphPad) and examined using Log-rank (Mantel-Cox) test with Bonferroni's correction to find the significant difference.

Oral infection of OBs in *B. mori* larvae

Oral infection was performed according to the previously described method[2]. ARIF-GFP OBs were produced in *B. mori* larvae, purified by centrifugation, resuspended in distilled water, and quantified using a hemocytometer. Fifth-instar larvae (within 24 h of molting) were orally inoculated with 5 µL of an OB suspension containing 2.5×10^6 OBs in 1% sucrose and returned to the artificial diet at 25°C. The infected larvae were dissected at specific time points for immunostaining of tissues.

Generation of a recombinant BmNPV expressing mCherry-fused GP64

To generate the mCherry::GP64-ARIF-GFP virus, we constructed the relevant plasmids using standard methods: PCR, restriction enzymes, and In-Fusion HD (Clontech). Here is an overview of the process: First, the ARIF-GFP genomic DNA was co-transfected with the pEPS-hsp-lacZ plasmid, which contains the BmNPV *polh* gene, its flanking sequences, and the *hsp70-lacZ* cassette in the upstream of the *polh* protein-coding sequence. Next, we isolated the hsp-lacZ-ARIF-GFP virus by identifying blue plaques expressing *β*-galactosidase using a plaque assay with agarose overlays containing 5-bromo-4-chloro-3-indolyl-*β*-D-galactoside. Subsequently, the Bsu36I-digested genomic DNA of hsp-lacZ-ARIF-GFP was cotransfected with the pEPS-mCherry::GP64 plasmid. This plasmid contains a fusion of mCherry with BmNPV *gp64*, including its original promoter sequence. mCherry was inserted just downstream of the GP64 signal peptide, fused with the N-terminus of the GP64 mature domain. Finally, we isolated the mCherry::GP64-ARIF-GFP virus by identifying white plaques using the abovementioned method.

The recombination was validated by PCR and DNA sequencing. All the primers used in these experiments are listed in Supplementary Table S1.

Quantification of viral genome replication and BV production in cultured cells

BmN cells were infected with BmNPVs at an MOI of 5 with three biological replicates. At 24 hpi, 5 µL of the culture medium containing released BVs was harvested, and BV production was determined by plaque assay[3]. Virus-infected cells were gently scraped with a rubber policeman, collected into microtubes, and intracellular total DNA was prepared as previously described[4]. qPCR was performed using KAPA™ SYBR FAST qPCR kit (Kapa Biosystems) with the primers rpolhF1 and rpolh R1 for virus genome and ago3_gF and ago3_gR for host genome (Supplementary Table S1)[4]. qPCR was performed using the StepOne real-time PCR system (Thermo Fisher Scientific), and the data were analyzed by StepOne Software v2.3 (Thermo Fisher Scientific).

Measurement of OB production in the larval cadaver

Newly-molted fifth-instar *B. mori* larvae were infected with hspGFP or hspGFP-ARIFD, and each cadaver was collected into a 50 mL conical tube and homogenized by vigorous pipetting in 10 mL distilled water. OB was counted using a hemocytometer to calculate total OB production per larva. The body weight of the larval cadaver was measured by an electronic balance. Finally, the total OB amount per gram was calculated.

Quantification of GFP fluorescence in cultured cells

BmN cells were infected with BmNPVs at an MOI of 5 with three biological replicates. At 24 hpi, GFP fluorescence was photographed using EVOS M5000 Cell Imaging System (Life Technologies). The fluorescent intensity of each cell was measured using the ParticleAnalyzer plugin in Fiji. The mean intensity value from > 500 cells in a single image was used as the fluorescence intensity of the image. Five images from each replicate were analyzed, and the mean value was used as the fluorescence intensity of the single replicate.

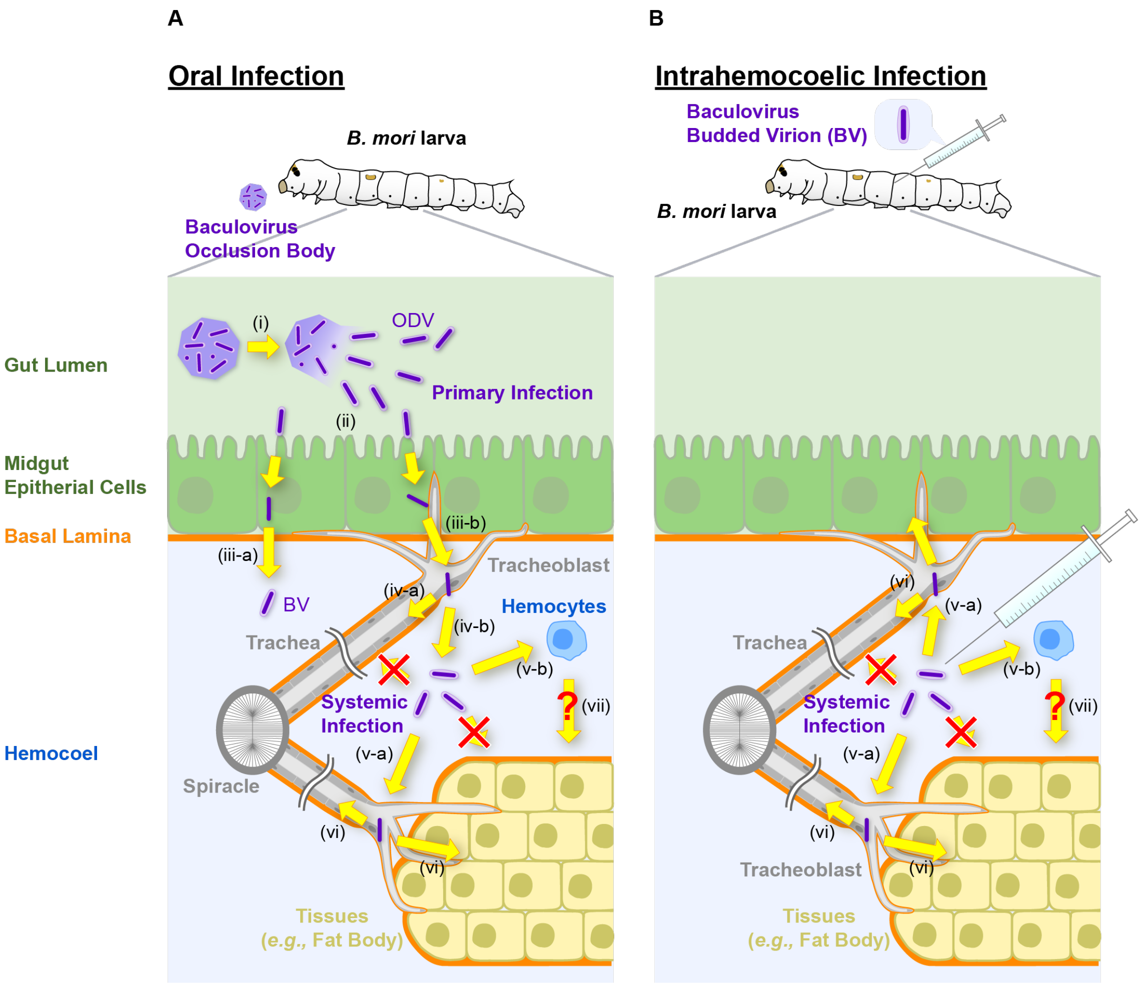

Supplementary Fig. S1. Previously proposed model of the baculovirus systemic infection. (**A**) Oral infection of viral occlusion bodies (OBs). Ingested OBs are disrupted by the alkaline pH of the host midgut lumen [i]. Occlusion-derived viruses (ODVs) are released into the gut lumen, establishing primary infection at midgut epithelial cells [ii]. Although baculovirus virions are too large to pass through the tiny pore of the basal lamina (BL), an early study implied direct crossing of the midgut BL by an unknown mechanism [iii-a][5,6]. Alternatively, baculoviruses bypass the midgut BL barrier by using the tracheoblasts (tracheal terminal cells that penetrate the BL of insect tissues) [iii-b][7]. Subsequently, the viral infection spreads in the trachea [iv-a], or the viruses release progeny budded viruses (BVs) into the hemolymph [iv-b]. BVs are presumably unable to transfer directly into the BL-covered tissues. Therefore, BVs in the hemolymph infect tracheoblasts throughout the host body [v-a] and then spread infection within the tissues [vi]. Exceptionally, BVs can directly infect hemocytes that do not have the BL barrier [v-b]. Hemocyte infection might be important for systemic tissue infection [vii][8], although its detailed mechanism is unknown. (**B**) Intrahemocoelic injection of BVs. After injection

into the hemolymph, BVs infect tracheoblasts and hemocytes [v], and then the viral infection spreads within the tissues like oral infection [vi, vii].

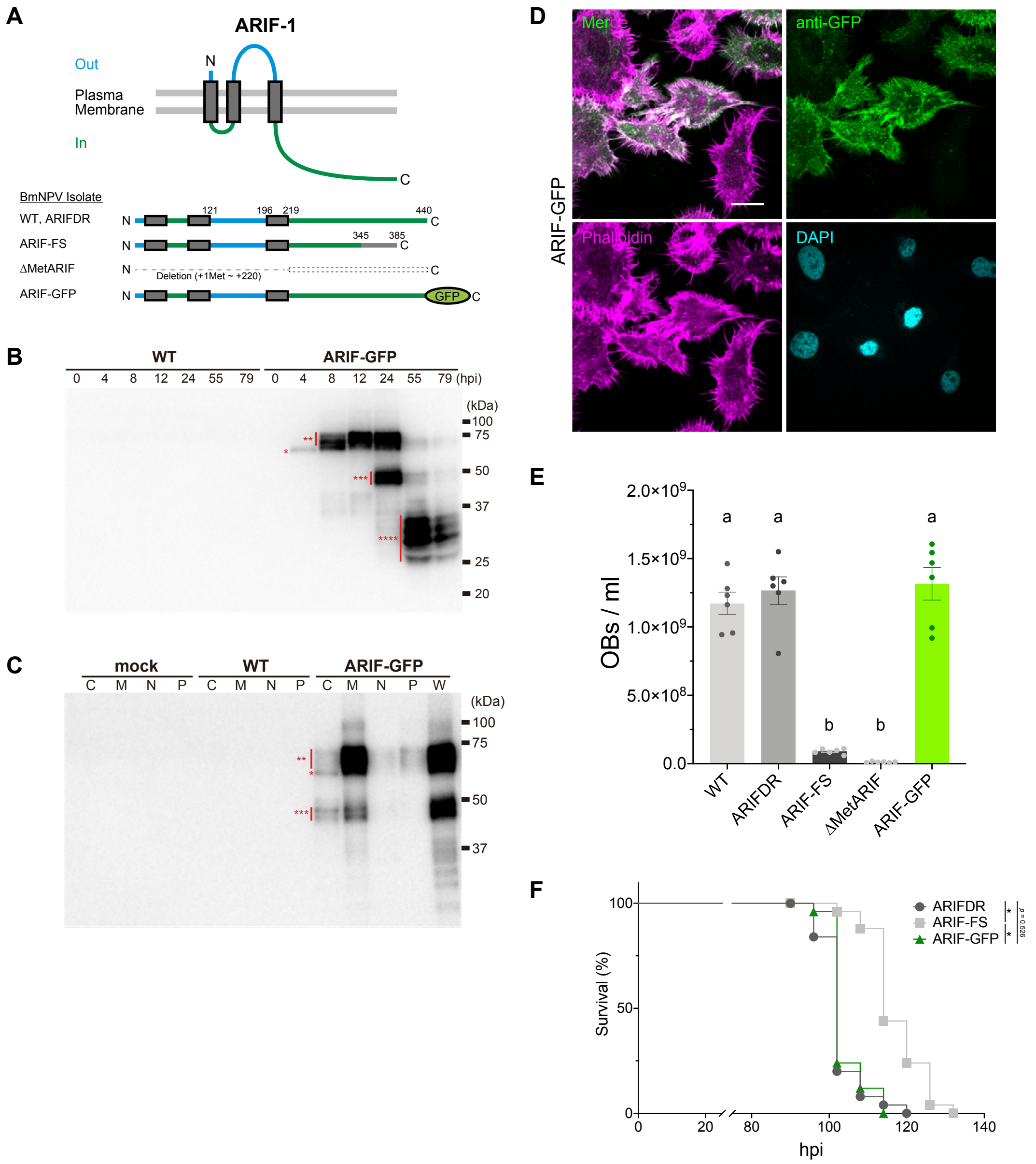

Supplementary Fig. S2. Characterization of GFP-fused ARIF-1. (**A**) Schematic image of ARIF-1 sequences in recombinant BmNPVs. (**B**–**C**) Expression of GFP-fused ARIF-1 in BmN cells. The cells were mock-infected or infected with WT or ARIF-GFP and collected at designated time points. Calculated molecular weight (Mw) of GFP-fused ARIF-1, 77.2 kDa. * and **, putative unphosphorylated and phosphorylated GFP-fused ARIF-1 (phosphorylation of AcMNPV ARIF-1 was reported in Dreschers et al. (2001)[9]); ***, 47–50 kDa extra bands, which might be a truncated ARIF-1 (calculated Mw: 53.9 kDa; a product of an mRNA transcribed from the viral late promoter at +552 nt of *arif-1*[10]; ****, 30–36 kDa extra bands, which possibly are degraded ARIF-GFP at the very late stage of infection. The differences between calculated Mw and actual band positions

are presumably due to the denaturation of protein samples at a mild condition (RT, O/N) to avoid aggregation. (**B**) Time-course expression of GFP-fused ARIF-1 in the whole cell extract. (**C**) Western blot analysis of subcellular-fractionated samples at 24 hpi. C, cytoplasmic extract; M, membrane extract; N, nuclear extract; P, pellet extract; W, whole cell extract. (**D**) Immunocytochemistry of GFP-fused ARIF-1 in *B. mori* cultured cells. BmN cells were infected with ARIF-GFP, fixed at 12 hpi, and stained with an anti-GFP antibody (green), rhodamine-phalloidin (magenta), and DAPI (cyan). Bar, 40μm. (**E**) The number of OBs released in the hemolymph of virus-infected *B. mori* larvae at 96 hpi. Data shown are mean ± SEM (n = 6). Different letters indicate statistically different groups ($p < 0.05$, one-way ANOVA with Tukey's multiple comparisons test). (**F**) Survival curves of ARIF-GFP-infected larvae. The median lethal times (LT_{50}) of ARIFDR, ARIF-FS, and ARIF-GFP are 102 h, 114 h, and 102 h, respectively (n = 30). *, $p < 0.0001$, Log-rank (Mantel-Cox) test with Bonferroni's correction.

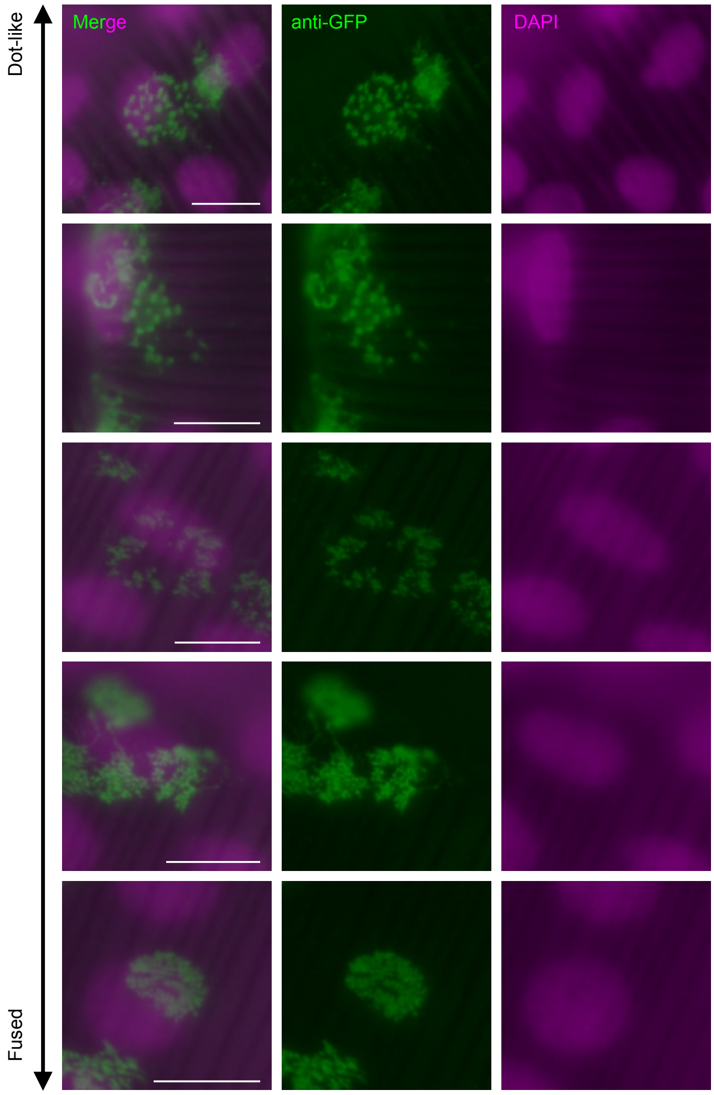

Supplementary Fig. S3. Remnants of invadosome-like structures. ARIF-GFP-infected trachea and hemocytes were fixed at 24 hpi and observed under a zoom microscope. The cell body of virus-infected hemocytes was gently crushed with a cover glass. Presumably, only the structure for attachment remained on the surface of the trachea. Invadosome-like structures showed a range of sizes and shapes, from more dot-like and separate ones (upper panels) to more fused and organized ones (lower panels). The top panels are the same pictures as Fig. 4D without brightness adjustment. Rabbit anti-GFP (green), GFP-fused ARIF-1; DAPI (magenta), dsDNA. Bar, 20 µm.

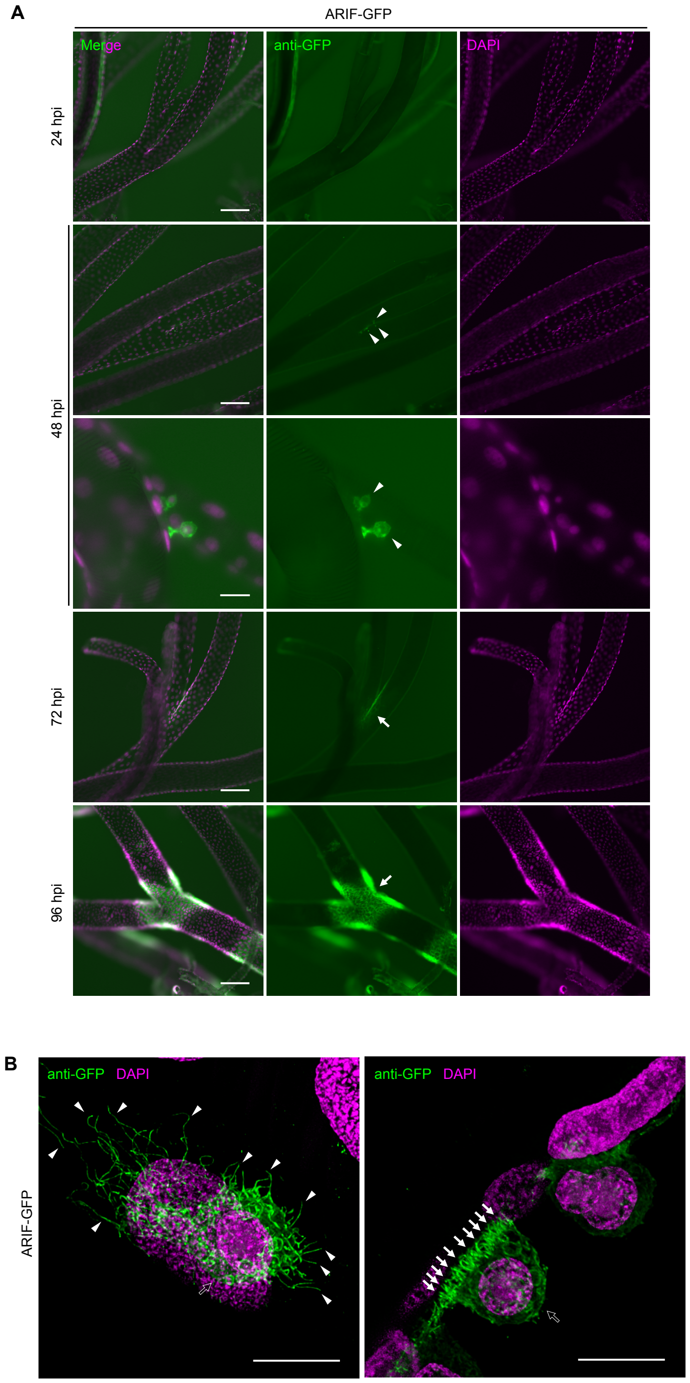

Supplementary Fig. S4. The invasive infection route via ARIF-1 also enhances systemic viral spread in oral infection. *B. mori* larvae were orally inoculated with ARIF-GFP OBs, and tracheal samples were dissected at 24, 48, 72, and 96 hpi for immunostaining. Anti-GFP, GFP-fused ARIF-1 (green); DAPI, dsDNA (magenta). Bar, 200 μm in (**A**) except for lower panels of 48hpi (10 μm); 10 μm in (**B**). (**A**) Spread of

infection in the trachea. No GFP-expressing cells were observed at 24 hpi. At 48 hpi, attachment of virus-infected hemocytes with GFP fluorescence was observed (white arrowheads). At 72 and 96 hpi, large infection foci were observed at the central tracheal region (white arrows), indicating infection via the invasive route. (**B**) Z-projection confocal images of attached hemocytes at 48 hpi with LIGHTNING deconvolution. White arrowheads and arrows, filopodia-like protrusions and invadosome-like structures, respectively.

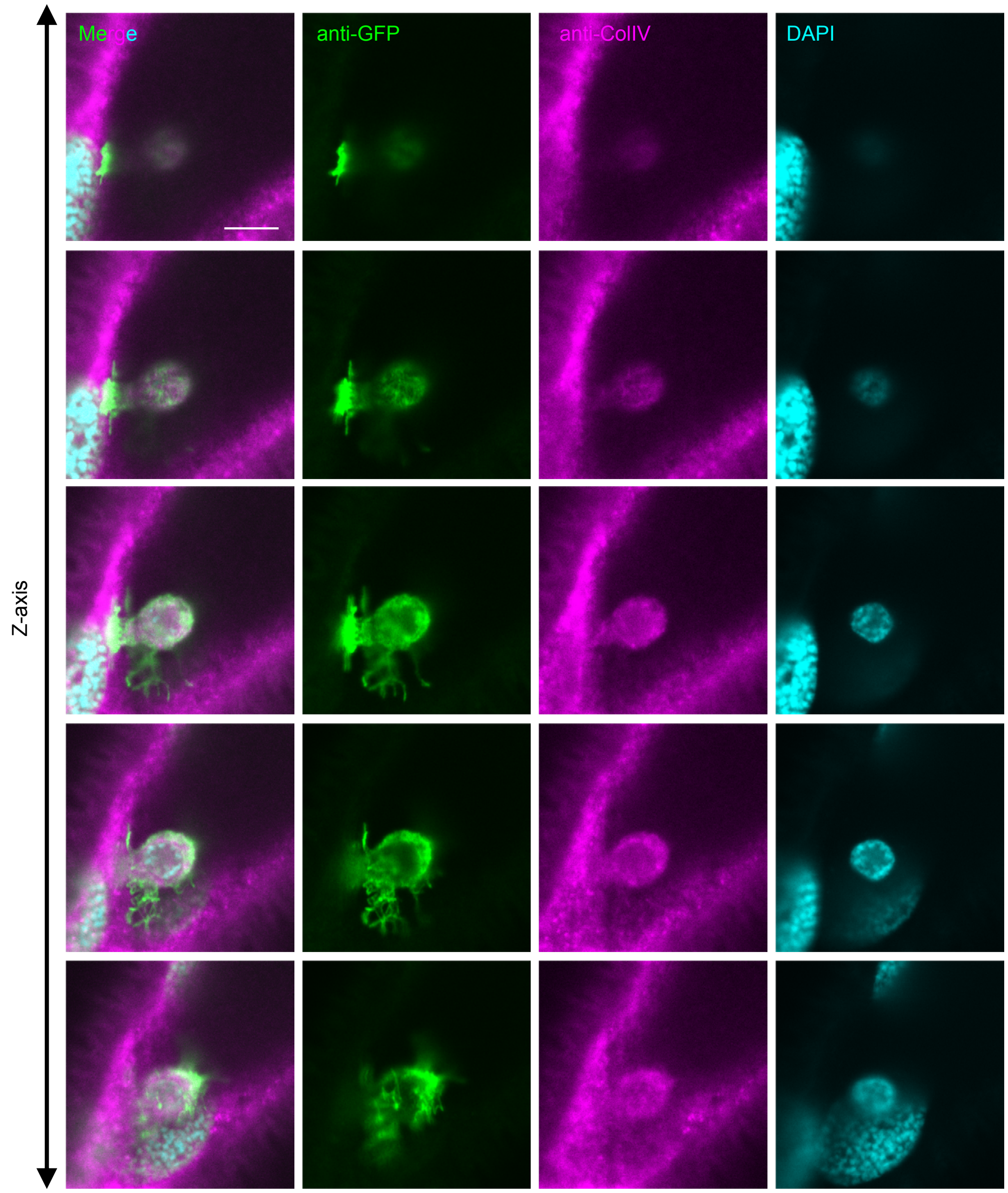

Supplementary Fig. S5. Serial confocal observation of the invadosome-like structure invading the trachea. The ARIF-GFP-infected hemocyte shown in Fig. 6C was serially observed under a confocal microscope at different focal depths. Anti-GFP (green), GFP-fused ARIF-1; anti-ColIV, collagen IV (magenta); DAPI (cyan), dsDNA. Bar, 10 µm.

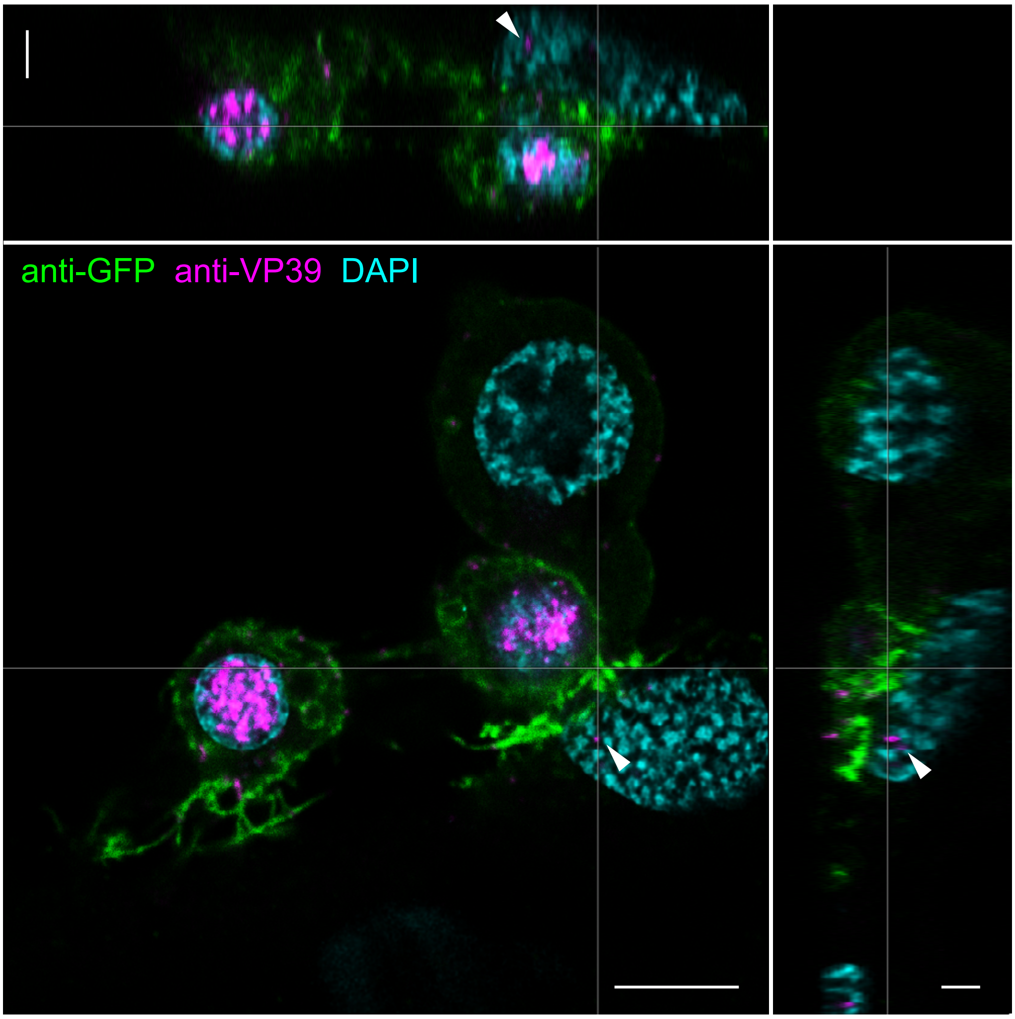

Supplementary Fig. S6. Orthogonal view of confocal microscope images from an ARIF-GFP-infected attached hemocyte. Immunostained ARIF-GFP-infected hemocytes (same as Fig. 7C) on the trachea at 24 hpi were observed under confocal microscopy with LIGHTNING deconvolution. Anti-GFP, GFP-fused ARIF-1 (green). Anti-VP39, VP39 (a major capsid protein, magenta). DAPI, dsDNA (cyan). White arrowheads, nucleocapsids in the nucleus of an adjacent tracheal cell. Bar, 10 µm.

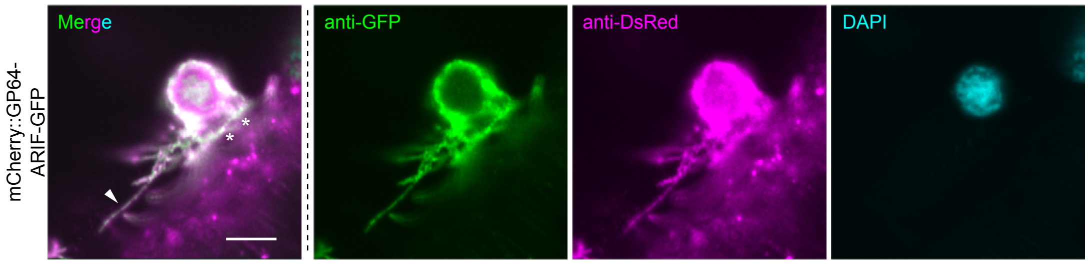

Supplementary Fig. S7. GP64 localization in the ARIF-1-derived structures. Immunostained mCherry::GP64-ARIF-GFP-infected hemocytes were observed under a confocal microscope at 24 hpi. A white arrowhead, a filopodia-like protrusion. Anti-GFP, GFP-fused ARIF-1 (green); anti-DsRed, mCherry-fused GP64 (magenta); DAPI, dsDNA (cyan). Bars, 10 µm. *, ARIF-1 aggregation at the interface between the hemocyte and the trachea (putative invadosome-like structures).

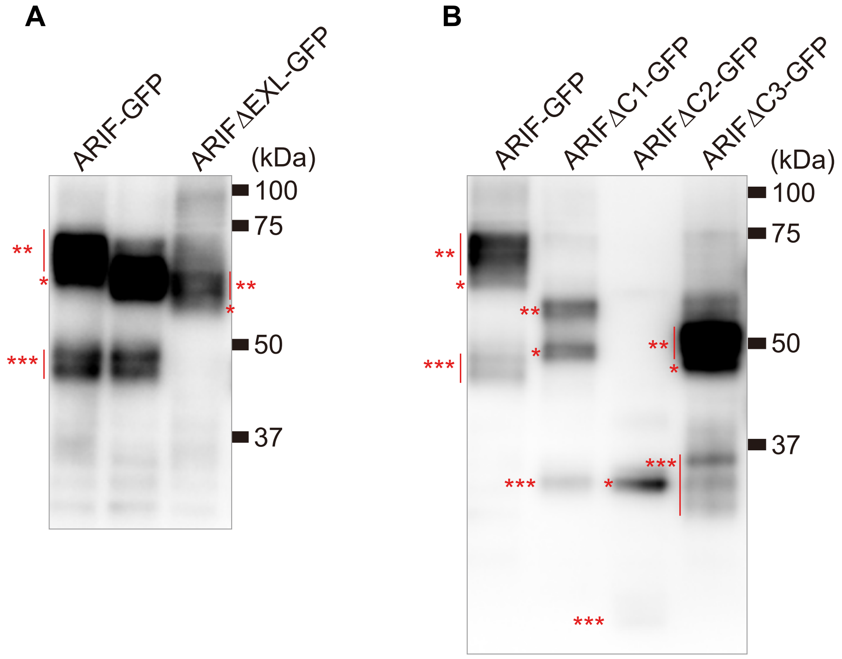

Supplementary Fig. S8. Expression of mutant ARIF-GFP proteins with partial deletions. BmN cells were infected with ARIF-GFP or its partial deletion mutants and collected at 24 hpi to obtain whole cell extracts. Calculated molecular weight (Mw): ARIF-GFP, 77.2 kDa; ARIFΔEXL-GFP, 69.5 kDa; ARIFΔC1-GFP, 66.7 kDa; ARIFΔC2-GFP, 53.5 kDa; ARIFΔC3-GFP, 64.0 kDa. * and **, putative unphosphorylated and phosphorylated ARIF-GFP and their partial deletion variants; ***, extra bands that might be a truncated ARIF-GFP (product of a mRNA transcribed from the viral late promoter at +552 nt of *arif-1*[10]) and its partial deletion variants. This viral late promoter is in the deleted region of ARIFΔEXL-GFP, thus the extra band was not detected in ARIFΔEXL-GFP. The differences between calculated Mw and actual band positions are presumably due to the denaturation of protein samples at a mild condition (RT, O/N) to avoid aggregation.

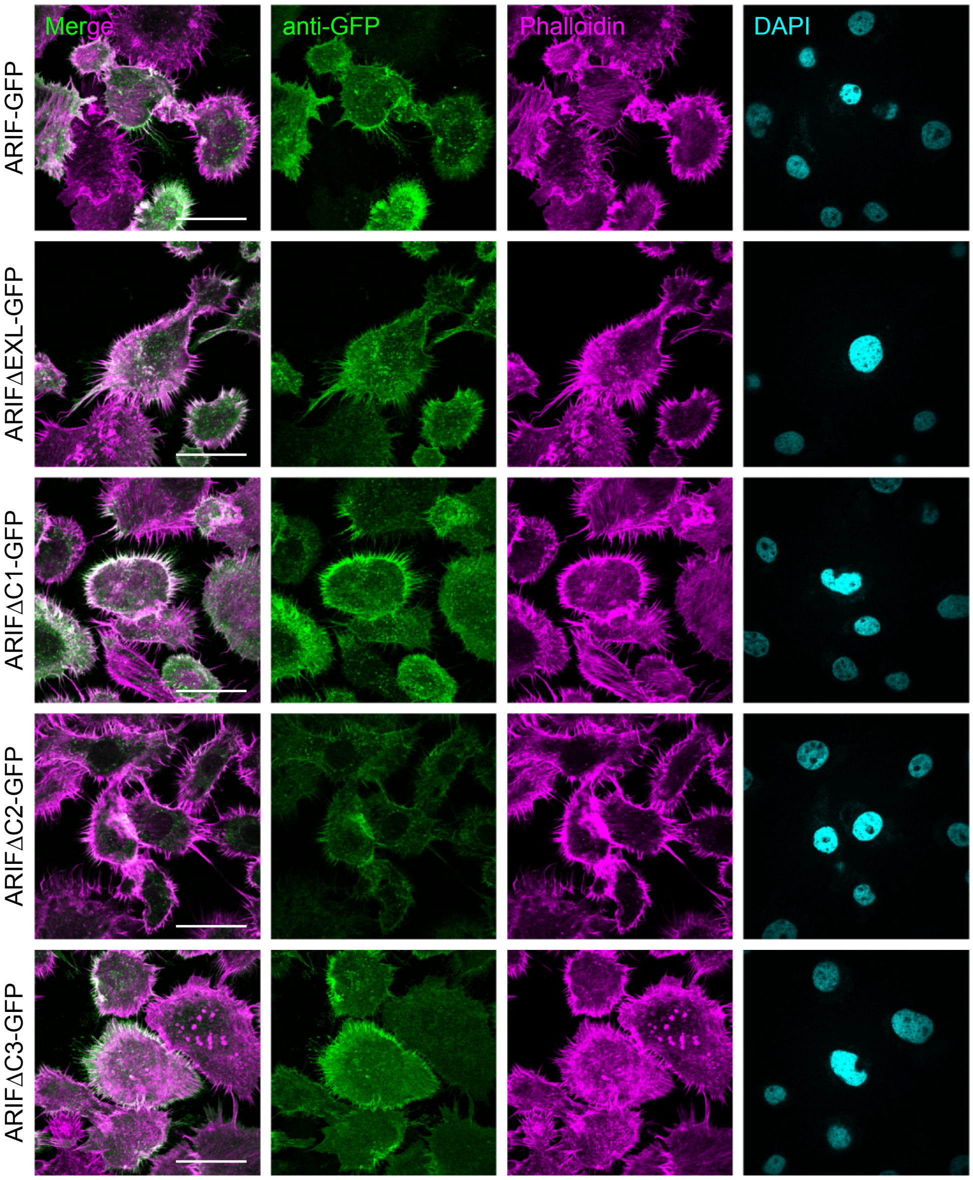

Supplementary Fig. S9. Subcellular localization of GFP-fused ARIF-1 proteins and its partial deletion variants in *B. mori* cultured cells. BmN cells infected with ARIF-GFP or its variants were fixed at 12 hpi and immunostained with anti-GFP antibody (green), rhodamine-phalloidin (magenta), and DAPI (cyan). The cells were then observed under a confocal microscope. Bar, 40 μm.

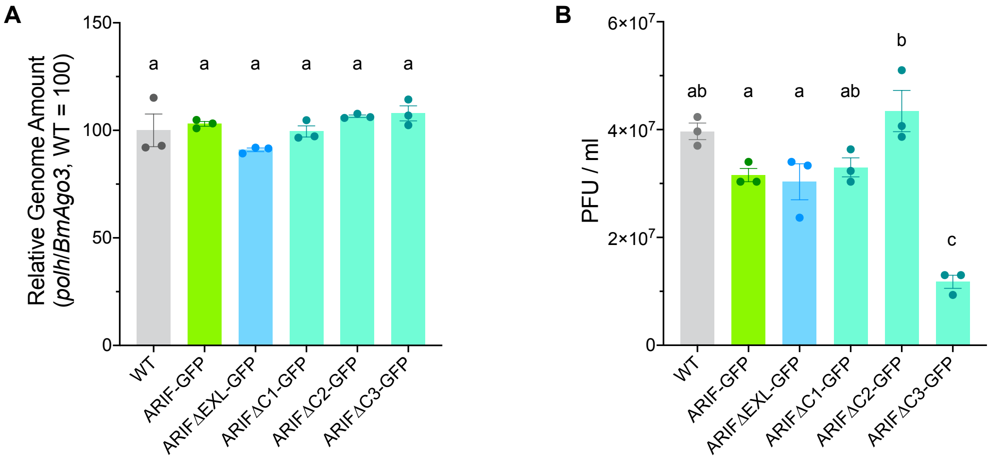

Supplementary Fig. S10. Virus genome replication and BV production of ARIF-GFP partial deletion variants in *B. mori* cultured cells. BmN cells were infected with each virus at an MOI of 5. At 24 hpi, virus-infected cells and culture supernatant were harvested for subsequent analyses. (**A**) Virus genome replication. The relative genome amount of each virus in infected cells was quantified by RT-qPCR. The *BmAgo3* gene was used for normalization by host genome amount. (**B**) BV production. Data shown in (**A**, **B**) are mean ± SEM (n = 3). Different letters indicate statistically different groups ($p < 0.05$, one-way ANOVA with Tukey's multiple comparisons test).

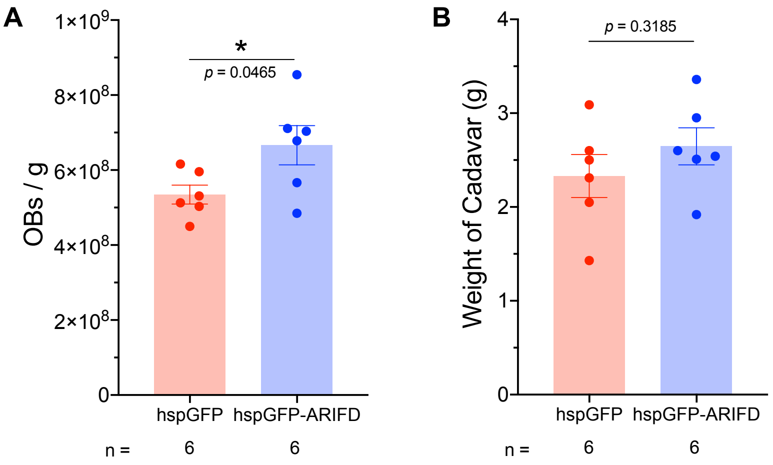

Supplementary Fig. S11. Total OB production in the larval cadaver. *B. mori* larvae were infected with hspGFP or hspGFP-ARIFD, and total OB production in each cadaver was calculated. (**A**) Total OB amount per gram in each cadaver. (**B**) The body weight of the larval cadaver. Data shown are mean ± SEM (n = 6). *, $p < 0.05$, unpaired *t*-test (two-tailed).

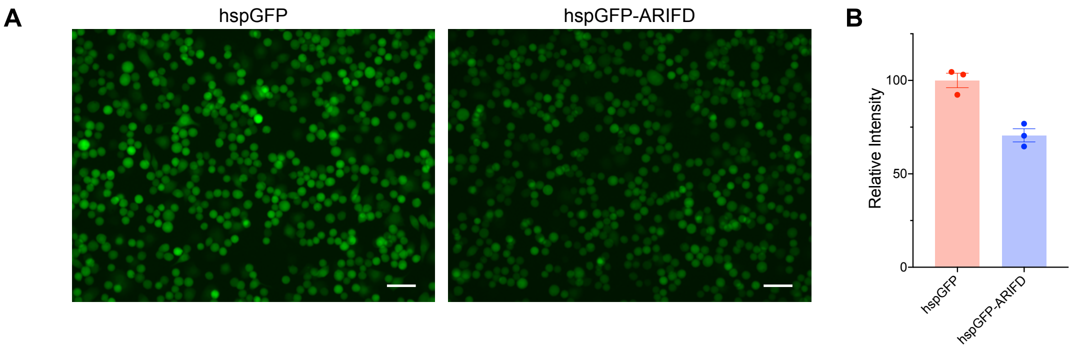

Supplementary Fig. S12. GFP expression of hspGFP and hspGFP-ARIFD viruses. BmN cells were infected with hspGFP or hspGFP-ARIFD at an MOI of 5, and GFP fluorescence was observed at 24 hpi under the EVOS imaging system. (**A**) Representative images of GFP fluorescence. Bar, 100 μm. (**B**) Fluorescence intensity. GFP fluorescence was quantified from the GFP fluorescent images using Fiji. Data shown are mean ± SEM (n = 3).

Table S1. List of primers.

Primer	Sequence (5′ to 3′)	Purpose
arif-1_ΔMet_inv_F	GCGTCGCAACATATACATAAAAAGGC	Construction of pcDNA-ΔMetARIF
arif-1_ΔMet_inv_R	TTTGAATATTTAATTCAACAACTAAGTAATGGC	Construction of pcDNA-ΔMetARIF
arif-1_BamHI_C-inv_F	ggatccTAGAAAATATTATATAAAGTATTATTTTTGATTATATAAAG	Construction of pcDNA-ARIF-BamHI
arif-1_C-inv_R	ATTATAAACGGATAGTTTTCTCTCTTTAAATTTACGC	Construction of pcDNA-ARIF-BamHI
EGFP_BamHI_F	AATGGATCCatggtgagcaagggcgaggagctgt	Construction of pcDNA-ARIF-GFP
EGFP-stop_BamHI_R	AATGGATCCcttgtacagctcgtccatgccgagagt	Construction of pcDNA-ARIF-GFP
arif1_ΔTMD3+_inv_F	AACTATTCCGTAATCATAATGGGCGTGCT	Construction of pcDNA-

		ARIFΔEXL-GFP
arif1_ΔEXD2only_inv_R	ATGACCATTTTTGAACACGTAGATTTGAAACG	Construction of pcDNA-ARIFΔEXL-GFP
arif1_Δ3_inv_F	ggatccATGGTGAGCAAGGGCGAGGAGCTGT	Construction of pcDNA-ARIFΔC1-GFP
arif1_Δ3_inv_R	TGTGGTACACAAAACTTTCTTGGGCACGTTGTA	Construction of pcDNA-ARIFΔC1-GFP
arif1_ΔC2_inv_F2	ggatccATGGTGAGCAAGGGCGAGGAGCTGTTCA	Construction of pcDNA-ARIFΔC2-GFP
arif1_ΔC2_inv_R3	tatgttgcgacgcatctctttTAATTGCACGTACAAATTCC	Construction of pcDNA-ARIFΔC2-GFP and pcDNA-ARIFΔC3-GFP
arif1_dC3/dC5_inv_F1	CcGGACGCGTGCACAATGTGCCAGCCTCCGTTG	Construction of pcDNA-ARIFΔC3-GFP

NotI-kozak-lacZ_F1	actGCGGCCGCaacaatcaaaATGGATAAAGTTTTCGGAATTCCAAGCTTGGCA	Construction of pEPS-hsp-lacZ
KpnI-lacZ_R1	actGGTACCttatttttgacaccagaccaactggtaatg	Construction of pEPS-hsp-lacZ
rpolhF1	GAACAAGAGGAGAAGCAATG	Quantification of virus genome amount
rpolh R1	TCCAGTTGGCGATTAACTTC	Quantification of virus genome amount
ago3_gF	TTTCTTAGTACACTCAAACG	Quantification of host genome amount
ago3_gR	CTCTCTTCGTAGAACATATC	Quantification of host genome amount

Supplementary References:

1. S. Katsuma, K. Mita, T. Shimada, ERK- and JNK-dependent signaling pathways contribute to *Bombyx mori* nucleopolyhedrovirus infection. *J. Virol.* ***81***, 13700–13709 (2007). doi:10.1128/JVI.01683-07
2. R. Kokusho, S. Katsuma, Loss of P24 from the Bombyx mori nucleopolyhedrovirus genome results in the formation of cuboidal occlusion bodies. *Virology* ***559***, 173–181 (2021). doi:10.1016/j.virol.2021.03.017
3. R. Kokusho, C.-X. Zhang, T. Shimada, S. Katsuma, Comparative analysis of budded virus infectivity of *Bombyx mandarina* and *B. mori* nucleopolyhedroviruses. *Virus Genes* ***43***, 313–317 (2011). doi:10.1007/s11262-011-0623-7
4. R. Kokusho, Y. Koh, M. Fujimoto, T. Shimada, S. Katsuma, Bombyx mori nucleopolyhedrovirus BM5 protein regulates progeny virus production and viral gene expression. *Virology* ***498***, 240–249 (2016). doi:10.1016/j.virol.2016.08.032
5. R. R. Granados, K. A. Lawler, *In vivo* pathway of *Autographa californica* baculovirus invasion and infection. *Virology* **108**, 297–308 (1981). doi:10.1016/0042-6822(81)90438-4
6. B. A. Federici, Baculovirus Pathogenesis in *The Baculoviruses* (ed. L. K. Miller), 33–56 (Springer New York, 1997). doi:10.1007/978-1-4899-1834-5
7. E. K. Engelhard, L. N. Kam-Morgan, J. O. Washburn, L. E. Volkman, The insect tracheal system: A conduit for the systemic spread of *Autographa californica* M nuclear polyhedrosis virus. *Proc. Natl. Acad. Sci. USA* **91**, 3224–3227 (1994). doi:10.1073/pnas.91.8.3224
8. B. A. Keddie, G. W. Aponte, L. E. Volkman, The pathway of infection of *Autographa californica* nuclear polyhedrosis virus in an insect host. *Science* **243**, 1728–1730 (1989). doi:10.1126/science.2648574
9. S. Dreschers, R. Roncarati, D. Knebel-Mörsdorf, Actin rearrangement-inducing factor of baculoviruses is tyrosine phosphorylated and colocalizes to F-actin at the plasma membrane. *J. Virol.* **75**, 3771–3778 (2001). doi:10.1128/JVI.75.8.3771
10. S. Katsuma, W.K. Kang, T. Shin-i, K. Ohishi, K. Kadota, Y. Kohara, T. Shimada, Mass identification of transcriptomic units expressed from the *Bombyx mori* nucleopolyhedrovirus genome. *J. Gen. Virol.* ***92***, 200–203 (2011). doi:10.1099/vir.0.025908-0
