# Supplementary material for: Baculoviruses remodel the cytoskeleton of insect hemocytes to breach the host basal lamina
Source: Commun Biol. 2025 Feb 26;8:268. doi: 10.1038/s42003-025-07579-x (PMC11865517; doi:10.1038/s42003-025-07579-x)
Supplement: Supplementary file 5 — Reporting Summary [file 42003_2025_7579_MOESM5_ESM.pdf]

nature portfolio

Corresponding author(s): Ryuhei Kokusho
Susumu Katsuma

Last updated by author(s): Jan 1, 2025

Reporting Summary

Nature Portfolio wishes to improve the reproducibility of the work that we publish. This form provides structure for consistency and transparency in reporting. For further information on Nature Portfolio policies, see our Editorial Policies and the Editorial Policy Checklist.

Statistics

For all statistical analyses, confirm that the following items are present in the figure legend, table legend, main text, or Methods section.

n/a	Confirmed	
☐	☒	The exact sample size (*n*) for each experimental group/condition, given as a discrete number and unit of measurement
☐	☒	A statement on whether measurements were taken from distinct samples or whether the same sample was measured repeatedly
☐	☒	The statistical test(s) used AND whether they are one- or two-sided<br>*Only common tests should be described solely by name; describe more complex techniques in the Methods section.*
☒	☐	A description of all covariates tested
☐	☒	A description of any assumptions or corrections, such as tests of normality and adjustment for multiple comparisons
☐	☒	A full description of the statistical parameters including central tendency (e.g. means) or other basic estimates (e.g. regression coefficient) AND variation (e.g. standard deviation) or associated estimates of uncertainty (e.g. confidence intervals)
☐	☒	For null hypothesis testing, the test statistic (e.g. *F*, *t*, *r*) with confidence intervals, effect sizes, degrees of freedom and *P* value noted<br>*Give P values as exact values whenever suitable.*
☒	☐	For Bayesian analysis, information on the choice of priors and Markov chain Monte Carlo settings
☒	☐	For hierarchical and complex designs, identification of the appropriate level for tests and full reporting of outcomes
☐	☒	Estimates of effect sizes (e.g. Cohen's *d*, Pearson's *r*), indicating how they were calculated

Our web collection on statistics for biologists contains articles on many of the points above.

Software and code

Policy information about availability of computer code

Data collection	Axio Zoom.V16 with objective lens PlanNeoFluar Z 2.3x/0.57 FWD 10.6 mm and ZEN software ver. 2.3 (ZEISS); Leica STELLARIS 5 WLL with 405 nm laser for DAPI and Leica Application Suite X Version 4.4.0.24861 (Leica); Nikon C1Si and EZ-C1 software Gold Version 3.60 build 770 (Nikon, Japan); FV10i ver.2.1.1.7 (OLYMPUS); EVOS M5000 Cell Imaging System (Life Technologies); SEM S-4800 (Hitachi Ltd., Japan); FLoid Cell Imaging Station (Thermo Fisher Scientific); Fiji software for macOS (x86_64); StepOne Software v2.3 (Thermo Fisher Scientific); Image Reader LAS-1000 Lite V1.31 (Fujifilm, Japan)
Data analysis	Leica Application Suite X Version 4.4.0.24861 (Leica); Fiji software for macOS (x86_64) with Particle Analyzer and Coloc2 (ver. 3.1.0) plugins; Prism 8 software (GraphPad); R (ver. 4.4.0) with the ggplot2 package (ver. 3.5.1), the effsize package (ver. 0.8.1), the dunn.test package (ver. 1.3.6); StepOne Software v2.3 (Thermo Fisher Scientific)

For manuscripts utilizing custom algorithms or software that are central to the research but not yet described in published literature, software must be made available to editors and reviewers. We strongly encourage code deposition in a community repository (e.g. GitHub). See the Nature Portfolio guidelines for submitting code & software for further information.

Data

Policy information about availability of data

All manuscripts must include a data availability statement. This statement should provide the following information, where applicable:

- Accession codes, unique identifiers, or web links for publicly available datasets
- A description of any restrictions on data availability
- For clinical datasets or third party data, please ensure that the statement adheres to our policy

All data are available in the main text or the Supplemental Information. Source Data are provided as a source data file.

Human research participants

Policy information about studies involving human research participants and Sex and Gender in Research.

Reporting on sex and gender	n/a
Population characteristics	n/a
Recruitment	n/a
Ethics oversight	n/a

Note that full information on the approval of the study protocol must also be provided in the manuscript.

Field-specific reporting

Please select the one below that is the best fit for your research. If you are not sure, read the appropriate sections before making your selection.

☒ Life sciences ☐ Behavioural & social sciences ☐ Ecological, evolutionary & environmental sciences

For a reference copy of the document with all sections, see nature.com/documents/nr-reporting-summary-flat.pdf

Life sciences study design

All studies must disclose on these points even when the disclosure is negative.

Sample size

For in vivo experiments in B. mori larvae (the concentration of circulating hemocytes, larval body weight, released OBs, survival curves, and total OB amount in larval carcasses) and in vitro experiments in BmN-4 cells (Western Blotting, quantifivation of viral genome amount, BV production, and GFP fluorescence quantification), sample sizes were selected based on the data obtained in previous studies.

Samples sizes of other experiments were determined based on the generation of convincing and compelling results. For analyzing infection foci, at least four picture was analyzed from each sample. For counting the number of attached hemocytes, five biological replicates were analyzed. For counting pH3-positive hemocytes, nine images each from two hemolymph samples were processed (155–689 cells in each image). For colocalization analysis, structures from at least three hemocytes were analyzed. For counting hemocytes with ARIF-1-derived structures, BL invasion, nuceleocapsid colocalization, and nucleocapsid invasion, at least 20 hemocytes were observed. For SEM analysis, two tissues from different individuals in each experimental group were examined.

The number of replicates is indicated in the figure legend and/or in the Methods section. The exact sample sizes and the values of each experiment were shown in Source Data.

Data exclusions

For analyzing the area of infection foci, infection foci with an area of less than 100 μm2 were excluded to remove noises.

For counting the number of attached hemocytes, we excluded hemocytes attached near the cutting sites and aggregated by Hemocytin from counting because they are presumably artifacts by dissection.

Replication

Experiments were repeated or replicated as described in the figure legends and the Methods section. All experiments shown could be reproduced as described.

Randomization

No randomization was performed. Experiments were performed by treating samples and controls side by side under indicated conditions.

Blinding

Blinding was not performed. Wherever possible, unbiased experimental procedures and data analysis were performed.

Reporting for specific materials, systems and methods

We require information from authors about some types of materials, experimental systems and methods used in many studies. Here, indicate whether each material, system or method listed is relevant to your study. If you are not sure if a list item applies to your research, read the appropriate section before selecting a response.

Materials & experimental systems

n/a	Involved in the study	
☐	☒	Antibodies
☐	☒	Eukaryotic cell lines
☒	☐	Palaeontology and archaeology
☐	☒	Animals and other organisms
☒	☐	Clinical data
☒	☐	Dual use research of concern

Methods

n/a	Involved in the study	
☒	☐	ChIP-seq
☒	☐	Flow cytometry
☒	☐	MRI-based neuroimaging

Antibodies

Antibodies used

Immunostaining:
The primary antibodies used were rabbit anti-GFP antibody (1:400; MBL, Japan, #598), mouse anti-GFP(15) monoclonal antibody (1:100–1:400; Santa Cruz, #sc-101525), and chicken anti-GFP antibody (1:200–1:500; abcam, # ab13970) for GFP, rabbit anti-Hemocytin antiserum (1:600–1:1000; provided by Dr. Ryoichi Sato) for Hemocytin, rabbit anti-Collagen IV (ColIV) antibody (1:50–1:200; abcam, #ab6586) for ColIV, rabbit anti-DsRed antibody (1:200; Clontech, #632496) for mCherry-fused GP64, mouse anti-VP39 antibody (1:100–1:400; provided by Dr. Loy E. Volkman) for VP39, and rabbit polyclonal anti-phospho Histone H3 (Ser10) antibody (anti-pH3) (1:200; Sigma-Aldrich, #06-570) for phosphorylated Histone H3. The secondary antibodies used were Alexa Fluor 488-conjugated goat anti-rabbit antibody (1:400; Invitrogen, #11070), Alexa Fluor 488-conjugated goat anti-mouse antibody (1:400; Invitrogen, #A11001), Alexa Fluor 488-conjugated goat anti-chicken antibody (1:200; Invitrogen, #A-11039), Alexa Fluor 546-conjugated goat anti-rabbit antibody (1:400; Invitrogen, #11071), or Alexa Fluor 546-conjugated goat anti-mouse antibody (1:400; Invitrogen, #11003).

Western Blotting:
The primary antibody used was rabbit polyclonal anti-GFP antibody (1:2,000; MBL, Japan; #598). The secondary antibody used was HRP-conjugated goat anti-rabbit IgG (1:5,000; Invitrogen; #656120).

Validation

Anti-Hemocytin antibody was produced in rabbit by Protein Purify Ltd. (Japan), and verified by Western blotting and immunostaining (Arai et al., Insect Sci., 2013; DOI:10.1673/031.013.12501).
Anti-VP39 antibody was produced in mouse and verified by Western blotting and immunostaining (Whitt & Manning, Virology, 1988; DOI:10.1016/0042-6822(88)90231-0).
All commercially available antibodies are produced in animals described above and verified by the manufacturers.

Eukaryotic cell lines

Policy information about cell lines and Sex and Gender in Research

Cell line source(s)	BmN-4 cells (Bombyx mori) are available from National BioResource Project Silkworm (Japan).
Authentication	BmN-4 cells were authenticated by qPCR amplification of the BmAgo3 gene and susceptibility for BmNPV.
Mycoplasma contamination	Not checked.
Commonly misidentified lines (See ICLAC register)	No commonly misidentified cell lines were used in this study.

Animals and other research organisms

Policy information about studies involving animals; ARRIVE guidelines recommended for reporting animal research, and Sex and Gender in Research

Laboratory animals	Bombyx mori (domesticated silkworm), fourth- or fifth-instar larvae, F1 hybrid Kinshu × Showa
Wild animals	n/a
Reporting on sex	We did not collect sex information because no significant sex-related difference has been reported in BmNPV pathogenicity on B. mori larvae.
Field-collected samples	n/a

Ethics oversight	This study does not require the ethical approval.

Note that full information on the approval of the study protocol must also be provided in the manuscript.
